# Methylcellulose-Encapsulated Magnesium-Substituted Biphasic Calcium Phosphate Granules for Local Drug Delivery in Bone Tissue Engineering: Modification for Prolonged Release and Antibacterial Behavior

**DOI:** 10.3390/polym17172422

**Published:** 2025-09-07

**Authors:** Daniil O. Golubchikov, Inna V. Fadeeva, Elena S. Trofimchuk, Katia Barbaro, Viktoriya G. Yankova, Iulian V. Antoniac, Valery I. Putlayev, Julietta V. Rau, Vicentiu Saceleanu

**Affiliations:** 1 Chemistry Department, Lomonosov Moscow State University, Leninskie Gory 1, 119991 Moscow, Russia; valery.putlayev@gmail.com; 2 Department of Materials Science, Lomonosov Moscow State University, Leninskie Gory 1, 119991 Moscow, Russia; 3 A.A. Baikov Institute of Metallurgy and Material Science, Russian Academy of Sciences, Leninsky 49, 119334 Moscow, Russia; fadeeva_inna@mail.ru; 4 Department of High-Molecular Compounds, Lomonosov Moscow State University, GSP-1, 1-3 Leninskiye Gory, 119991 Moscow, Russia; trofimchuk@vms.chem.msu.ru; 5 Istituto Zooprofilattico Sperimentale Lazio e Toscana “M. Aleandri”, Via Appia Nuova 14111, 00178 Rome, Italy; katia.barbaro@izslt.it; 6 Department of Analytical, Physical and Colloid Chemistry, Institute of Pharmacy, Federal State Autonomous Educational Institution of Higher Education I.M. Sechenov First Moscow State Medical University of Ministry of Health of the Russian Federation (Sechenovskiy University), Trubetskaya 8, Build. 2, 119048 Moscow, Russia; yankova_v_g@staff.sechenov.ru; 7 Faculty of Material Science and Engineering, National University of Science and Technology Politehnica Bucharest, 313 Splaiul Independentei, District 6, 060042 Bucharest, Romania; iulian.antoniac@upb.ro; 8 Academy of Romanian Scientists, 54 Splaiul Independentei, 050094 Bucharest, Romania; 9 Istituto di Struttura Della Materia, Consiglio Nazionale Delle Ricerche, ISM-CNR, Via del Fosso del Cavaliere 100, 00133 Rome, Italy; 10 Faculty of Medicine, University Lucian Blaga Sibiu, 2A Lucian Blaga Street, 550169 Sibiu, Romania; vicentiu.saceleanu@ulbsibiu.ro

**Keywords:** bone tissue engineering, biphasic calcium phosphate, magnesium-substituted calcium phosphate, antibacterial drug delivery, antimicrobial properties, methylcellulose composite

## Abstract

Bone tissue restoration requires biomaterials, which combine osteoinductivity and the capability to prevent surgical site infections. Magnesium-substituted biphasic calcium phosphate (Mg-BCP) represents a promising solution, as magnesium substitution increases the biodegradation rate of calcium phosphate ceramics and provides inherent antibacterial properties. This study aimed to achieve wet precipitation synthesis of magnesium-substituted (1–10 mol%) biphasic calcium phosphate and to evaluate its drug delivery potential and antibacterial performance. Porous Mg-BCP granules were fabricated via the gelation of Mg-BCP suspension in sodium alginate followed by polymer removal. Drug delivery potential was evaluated using methylene blue as a model compound, with methylcellulose encapsulation implemented to ensure prolonged release. Magnesium content directly ruled the phase composition: low concentrations (1%) favored hydroxyapatite phase prevalence, while higher concentrations led to the β-tricalcium phosphate formation. Further assessment of drug delivery potential revealed that direct drug loading resulted in burst release, whereas methylcellulose encapsulation successfully enabled prolonged drug delivery. Mg-5BCP formulation demonstrated significant antimicrobial activity with growth inhibition of 17.7 ± 4.1% against *C. albicans*, 20.8 ± 7.0% against *E. faecalis*, and 12.9 ± 7.5% against *E. coli*. Therefore, Mg-5BCP–methylcellulose composite granules present a versatile platform for antibacterial drug delivery for bone tissue engineering applications.

## 1. Introduction

Large bone defects may result from different injuries and, in some cases, exceed the critical size, which requires the insertion of an implant via a surgical intervention, aimed at providing mechanical integrity and structural support [1,2]. In addition to satisfactory mechanical properties, materials for bone tissue engineering should be capable of osteoconduction, which can be ensured by the fabrication of porous architectures by 3D printing and osteoinduction to ensure optimal new bone formation and the vascularization of the defect [3,4]. Another important feature that determines the efficiency of the bone healing process is the biodegradation rate, which should be tailored to the target defect [5].

Conventional ceramic materials based on hydroxyapatite (HAp) lacked resorbability, which led to the development of ceramic scaffolds, fabricated from more soluble compounds, including β-tricalcium phosphate (β-TCP), octacalcium phosphate, or calcium pyrophosphate [6,7,8]. The adjustment of the biodegradation rate can also be achieved via the implementation of biphasic ceramics (BCP: HAp/β-TCP) with different Ca/P ratios [9].

Further cationic doping of calcium phosphate materials may further adjust the biodegradation rate. Attention should be mainly attributed to magnesium, which participates in many metabolic processes during bone regeneration [10]. The introduction of magnesium was reported to stimulate osteogenic differentiation with further osteoblast proliferation. Moreover, the addition of Mg^2+^ has been shown to promote the mineralization of the extracellular matrix [11,12,13]. The vascularization of the inserted scaffold can also be improved by calcium phosphates doped with magnesium, as Mg^2+^ ions promote the production of vascular endothelium growth factor [13,14].

In addition to improving osteoinductivity, cationic doping can address the issue of surgical site infections, which affect up to 5% of patients following the restoration of fractures. The main pathogens related to periprosthetic joint infection are Gram-positive *Staphylococcus aureus* (*S. aureus*) and Gram-negative *Pseudomonas aeruginosa* (*P. aeruginosa*) and *Escherichia coli* (*E. coli*) [15]. There were several recent studies regarding the assessment of the antibacterial performance of cationic-doped calcium phosphates. Sr-doped β-TCP moderately impeded the growth of *E. coli* and *S. aureus* and prevented biofilm formation [16], while Co-doped β-TCP sufficiently inhibited the growth of *C. albicans* [17]. Ni-doped HAp also showed modest inhibition activity of *E. coli* growth [18]. Moreover, anionic dopants, such as borates and silicates, also acted as bacterial growth inhibitors [19,20]. Despite the antibacterial efficiency, these elements showed certain cytotoxicity even at relatively low contents [21,22,23]. In contrast, while magnesium-doped calcium phosphates showed similar antibacterial efficiency, they have not showed notable cytotoxic effects [24,25]. Further enhancement of the antibacterial performance can be achieved by absorbing antibiotic molecules on the surface of ceramic particles. While this has been proven to be effective [26], the encapsulation of particles into the polymeric matrix can provide more stable release of antibiotic molecules.

There have been several synthesis approaches aimed at obtaining doped biphasic calcium phosphates/silicates, including wet precipitation, the sol–gel method, the hydrothermal method, and spray-drying [11,27,28,29,30]. As the Ca(Mg)/P ratio of the ceramic scaffold plays a vital role in the bone tissue restoration, affecting the osteogenic performance and biodegradation rate, many studies focused on the synthesis of Mg-substituted BCP with a target ratio, which should be in the range of HAp/β-TCP = 30/70–70/30 to achieve stable biodegradation and new bone formation [31], while pure phases showed lower osteogenic potential [32]. Nevertheless, the introduction of cations, such as Sr^2+^ and Mg^2+^, was shown to affect the crystallization process during the wet precipitation, therefore affecting the final Ca/P ratio. In this paper, we focused on this effect to reveal the influence Mg^2+^ concentration on the BCP composition.

Therefore, this study focused on synthesizing magnesium-substituted biphasic calcium phosphate (HAp/β-TCP = 40/60) by the wet precipitation method by mediating the magnesium content with a further formation of porous spherical granules for assessing the drug delivery potential. We compared direct drug loading (methylene blue) and methylcellulose encapsulation. Moreover, the raw magnesium-substituted biphasic calcium phosphate (Mg-BCP) powders showed promising results in inhibiting the growth of *C. albicans*, *E. faecalis*, and *E. coli*.

## 2. Materials and Methods

### 2.1. Materials

Powders of calcium nitrate (Ca(NO_3_)_2_, analytical grade, Labtech, Moscow, Russia), magnesium nitrate (Mg(NO_3_)_2_), analytical grade, Labtech, Moscow, Russia), and ammonium phosphate dibasic ((NH_4_)_2_HPO_4_, analytical grade, Sigma-Aldrich, St. Louis, MO, USA) were used as precursors for the synthesis of magnesium-substituted non-stoichiometric hydroxyapatite. Sodium alginate (Labtech, Moscow, Russia) was used for the formation of BCP granules. Methylcellulose (LLC Polycon, Moscow, Russia, Mw = 50 kDa) was used for encapsulating methylene blue (Reachim, Moscow, Russia) in the BCP granules.

### 2.2. Synthesis of Magnesium-Substituted Non-Stoichiometric Hydroxyapatite

Synthesis of magnesium-substituted non-stoichiometric hydroxyapatite (MgHAp) was performed via the precipitation from the water solution of precursors with different magnesium content: 1 mol% (Mg-1BCP), 5 mol% (Mg-5BCP), and 10 mol% (Mg-10BCP). In brief, water solution of ammonium phosphate dibasic (0.6 M) was added by drops to the water solution of calcium nitrate and magnesium nitrate (1 M) (in the corresponding content) according to the following Equation (1):

(10 − x)Ca(NO_3_)_2_ + xMg(NO_3_)_2_ + 6 (NH_4_)_2_HPO_4_ + 8NH_4_OH → Ca_10−x_Mg_x_(PO_4_)_6_(OH)_2_ + 20NH_4_NO_3_ + 6H_2_O (1)

where x corresponds to the content (x = 0.01 for Mg-1BCP, 0.05 for Mg-5BCP, and 0.1 for Mg-10BCP) of magnesium in the solution. The synthesis was conducted in a chemical reactor with an overhead stirrer and dropping funnel. After the initial precipitation, the stirring process continued for 1 h with subsequent aging for 24 h. Then, the precipitate was separated by filtration on a Buchner funnel, washed with cold distilled water, and dried in a heating chamber at 110 °C for 15 h. The obtained powders were calcined at 400 °C to remove the residues of ammonium nitrate and water. For further production of biphasic calcium phosphate powders from non-stoichiometric hydroxyapatite, which occurs after 700 °C [33], powders were heated up to 900 °C in a chamber furnace with a SiC heater (SNOL, Riga, Latvia) in a corundum crucible.

### 2.3. Formation of BCP Granules and Loading of Model Antimicrobial Agent

The granulation was performed for synthesized powders after a 400 °C calcination. The suspension of MgHAp in a sodium alginate (SA) water solution was prepared with 0.5–2 MgHAp/SA ratio. The suspension was added by drops to the 5% barium nitrate water solution, which led to the formation of spherical granules. Obtained granules were filtered and sintered in a chamber furnace with a SiC heater (SNOL, Riga, Latvia) in a corundum crucible at 1100 °C for 2 h with a heating rate of 5 °C/min, which led to the formation of Mg-BCP [34].

The granules were impregnated with the model antibiotic methylene blue (MB) 1 wt% water solution after preliminary degassing at a pressure of 10^−2^ mm Hg. After impregnation for 30 min, the granules were dried at room temperature for 16 h. To achieve long-term release, the encapsulation with methylcellulose (MC) was implemented. MC was preliminary dissolved in the MB solution at a concentration of 5 wt%, and the obtained solution was used for Mg-BCP granule impregnation.

### 2.4. Physical and Chemical Characterization

The phase composition of the magnesium-substituted biphasic calcium phosphate samples after the synthesis was determined using X-ray powder diffraction (XRD) analysis using a Rigaku D/Max-2500 diffractometer (Rigaku Corporation, Tokyo, Japan) implementing a rotating anode (Cu–Ka radiation, λ = 0.15418 nm) and angle interval 2θ: from 10° to 60° (step 2θ − 0.02°). To execute the phase analysis, we utilized the ICDD PDF2 database.

Infrared spectra have been obtained using Spectrum 3 spectrometer (Perkin Elmer, Waltham, MA, USA) in attenuated total reflectance mode in the range of wavenumbers from 520 to 4000 cm^−1^ with the Universal ATR accessory (diamond/KRS-5 crystal).

To measure the specific surface area of the powders, the low-temperature adsorption–desorption method of Brauner–Emmett–Teller (BET) was used (“TriStar-3000” analyzer, Micromeritics, GA, USA).

Scanning electron microscopy (SEM) images of the obtained Mg-5BCP granules were collected by NVision 40 microscope (Carl Zeiss, Jena, Germany) at the accelerating voltage of 5 kV in a secondary electron imaging mode (SE2 detector). A chromium coating (≤10 nm thick) was deposited onto the surface of the ceramic sample using a Quorum Technologies Q150T ES spraying system (London, UK).

Biodegradation rate was assessed by monitoring the concentration of Ca^2+^ ions in the stated TRIS buffer by ICP-MS (ICP Optima 5300 ICP Optical Emission Spectrometer (Perkin-Elmer, Waltham, MA, USA)).

To evaluate the methylene blue release from Mg-5BCP granules and MC-encapsulated Mg-5BCP composite, samples containing methylene blue were immersed in a physiological solution and kept at 37 °C. For quantitative determination of methylene blue released from the ceramic granules and composite, 5 mL of the solution was collected 1, 3, and 5 days after immersion of the granules or composite with methylene blue in a physiological solution.

The concentration of methylene blue in the solution was determined photometrically using a UV 6900 spectrophotometer (Shanghai Mapada, Shanghai, China) at a wavelength of 662 nm using a calibration graph constructed in the concentration range from 0.001 to 0.1 mg/L.

Rheological properties of methylcellulose solutions in 0.9% aqueous NaCl solution with concentrations from 1 to 7 wt% were studied on a RheolabQC rheometer (Anton Paar, Graz, Austria) using a CC39/T200/XL/SS measuring system (cup + spindle) in the shear rate range from 6 to 230 s^−1^. Dynamic viscosity was determined at temperatures of 25 °C and 37 °C. Temperature was controlled (±0.2 °C) using a C-LTD80/QC thermostatic flow cell (Anton Paar, Graz, Austria). The device was calibrated according to the GSO REV-1000-EK standard (OOO Ekros, Saint-Petersburg, Russia).

### 2.5. Antimicrobial Tests

The antimicrobial properties of β-TCP and Mg-5BCP were evaluated using four bacterial strains—*Escherichia coli*, *Staphylococcus aureus*, *Pseudomonas aeruginosa*, and *Enterococcus faecalis*—and one fungal strain, *Candida albicans*. Prior to use, the powders were sterilized by autoclaving at 121 °C for 20 min.

Microorganisms were cultured in Brain Heart Infusion (BHI) broth (DIFCO, Sparks, NV, USA) containing 1 mg/mL of TCP or Mg-BCP and incubated for 24 h under their respective optimal growth conditions with gentle shaking: 37 °C for bacterial strains and 28 °C for *C. albicans*. For each assay, a positive control was prepared by culturing the microorganisms in BHI without powders. All experiments were performed in triplicate. Microbial growth was quantified by measuring the optical density at 600 nm using a BioPhotometer (Eppendorf, Hamburg, Germany).

### 2.6. Statistical Analysis

Growth rate data were expressed as the mean ± standard deviation (SD) of three independent replicates and statistically analyzed. Multiple comparisons of mean values were performed using Dunnett’s test (OriginPro 2016, ver. b9.3, OriginLab). The *p*-values were reported as follows: *p* < 0.05 *, *p* < 0.01 **, and *p* < 0.001 *** compared to the control.

## 3. Results and Discussion

Synthesis conditions have a major effect on the Ca/P ratio in non-stoichiometric hydroxyapatite [9,35]. Moreover, the introduction of different ions, including magnesium, may lead to the stabilization of amorphous calcium phosphate, which can further cause the formation of HAp/ β-TCP ceramics. After the precipitation of non-stoichiometric hydroxyapatite from the water solution, we obtained three powders with different magnesium contents in the range of 1–10%, which were further subjected to sintering at 900 °C.

**Figure 1:**
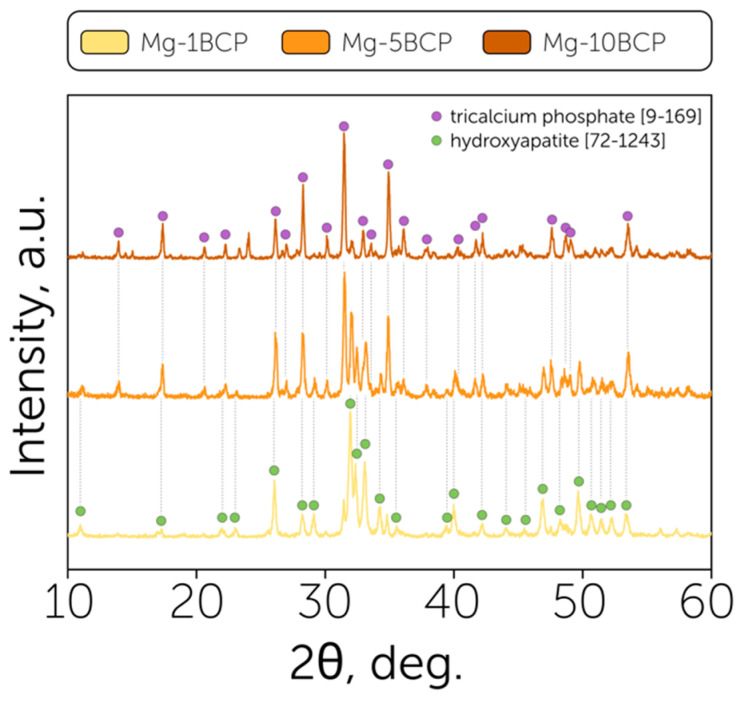
Powder characterization: phase composition of the powder obtained by sintering of the magnesium-substituted non-stoichiometric HAp with different Mg contents (Mg-1BCP, Mg-5BCP, Mg-10BCP) with β-tricalcium phosphate [9-169] and hydroxyapatite [72-1243] characteristic peaks.

The phase composition of the obtained phases is presented in Figure 1. Two main phases were presented in the obtained powder samples: β-tricalcium phosphate and hydroxyapatite, attributed to the ICDD PDF-2 [9-169] and [72-1243] cards, correspondingly. The slight shifts in peaks from the positions indicated in the ICDD database were attributed to the introduction of smaller Mg^2+^ ions to the Ca^2+^ positions.

The primary phase obtained with a 1% Mg substitution was hydroxyapatite with a negligible presence of tricalcium phosphate, while 10% Mg substitution led to β-TCP as the main phase, which can be explained by the stabilization of amorphous calcium phosphate by the Mg^2+^ ions during the synthesis according to the previously reported mechanism [36]. The regulation of the Ca/P ratio by the Mg content allowed target biphasic powders (ω(HAp) = 43%, ω(β-TCP) = 57%) to be obtained by introducing the intermediate content of magnesium (5%), which was considered for the further formation of granules and antibacterial examination. The analysis of the quantitative composition of biphasic calcium phosphates has been realized by the corundum numbers method.

**Figure 2:**
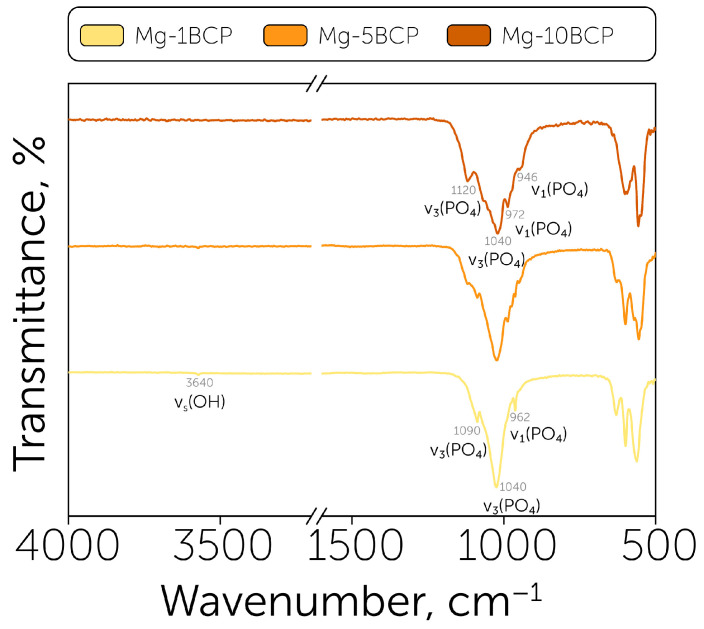
Powder characterization: FTIR spectra of Mg-1BCP, Mg-5BCP, and Mg-10BCP powders with indicated characteristic line positions.

The FTIR spectra showed the significant appearance of HAp in the sample obtained with a 1% magnesium content (Figure 2). The Mg-1BCP spectrum had a relatively strong 1090 cm^−1^ line, which can be attributed to the ν_3_ PO_4_ line position characteristic for hydroxyapatite. In contrast, the spectrum of Mg-10BCP had a β-TCP-specific shoulder line appear at this position. Moreover, the Mg-1BCP spectrum had a ν_1_ PO_4_ line at 962 cm^−1^ and a v_s_ (OH) at 3640 cm^−1^. The 972 cm^−1^ and 946 cm^−1^ bonds occurred specifically for the Mg-10BCP sample and corresponded to the ν_1_ PO_4_ line in β-TCP [37]. For the BCP sample, both HAp- and β-TCP-specific lines appeared, which correlated with the dependances revealed by XRD.

**Figure 3:**
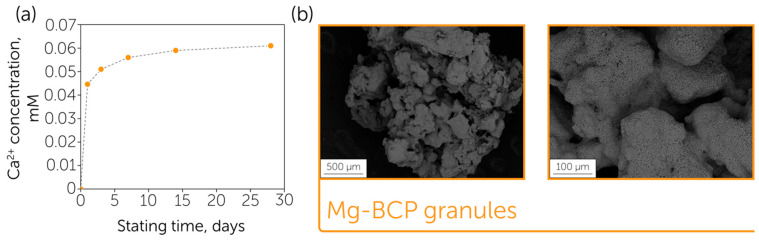
BCP granule characterization: (**a**) Ca^2+^ release profile during the dissolution of Mg-5BCP granules in TRIS buffer; (**b**) SEM images of Mg-5BCP ceramic granules.

A further part of the research was focused on the formation of porous Mg-5BCP granules and their modification. Sodium alginate is widely known for the formation of cross-linked gels with polyvalent metal ions, which can be further utilized for the fabrication of ceramic granules with a uniform size distribution [38]. In particular, the initial Mg-5BCP powder was immersed in the sodium alginate solution. Further 5% Ba(NO_3_)_2_ water solution addition led to the formation of granules with a spherical shape. Heat treatment at 1100 °C led to the SA burning out and the formation of porous ceramic granules with sizes up to 10 µm. It is worth noting that a consistent elimination of gaseous SA decomposition products led to the formation of an open and interconnected system of pores, essential for the loading of different bioactive molecules (Figure 3b). The open porosity (macropores) estimated from the SEM images was 29.1 ± 2.6%, despite the nanoporosity being modest as the cumulative volume of pores was 0.01 cm^3^/g according to the nitrogen adsorption analysis. The calculated BET surface area was 3.6786 m^2^/g. It is worth noting the potential limitations of open porosity calculated from SEM images, which do not consider the surface topography, with this also being a local method.

Another important aspect of the BCP granule performance was the biodegradation behavior, which was assessed in the experiment conducted using physiological solution containing a buffer, which maintained a pH close to neutral (TRIS, pH = 7.4). When Mg-5BCP granules were placed to the TRIS buffer, two processes occurred simultaneously: the granules were dissolving, which resulted in the release of Ca^2+^ ions to the solution and an increase in their concentration, which was accompanied by the precipitation of hydroxyapatite on the surface of BCP granules that led to the decrease in the concentration of calcium ions in the media. When the dynamic equilibrium was reached, the concentration of calcium ions in the solution remained unchanged (Figure 3a).

**Figure 4:**
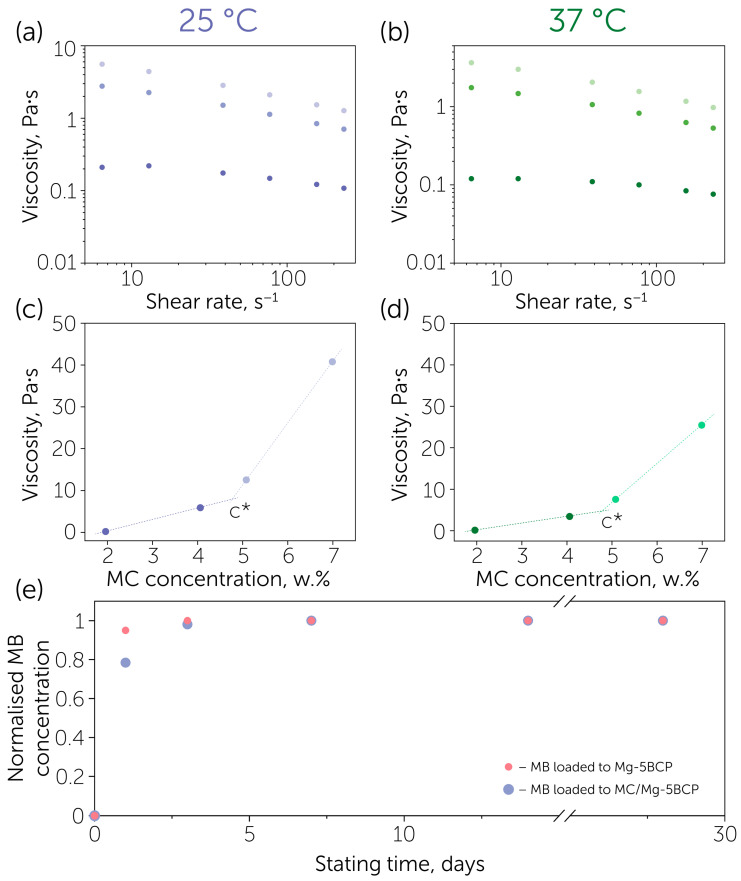
Approach to prolong the release profile: (**a**,**b**) dynamic viscosity of 2% (dark purple at 25 °C, dark green at 37 °C), 4% (purple at 25 °C, green at 37 °C), 5% (light purple at 25 °C, light green at 37 °C) methylcellulose water solution (0.9% NaCl) at 25 °C and 37 °C; (**c**,**d**) viscosity dependance from the methylcellulose concentration graphs at 25 °C and 37 °C (dark dots—diluted state, light dots—concentrated state), C*—transition point between the states of polymer chains in the solution; (**e**) release profile of methylene blue from raw Mg-5BCP granules (red dots) and MC-encapsulated Mg-5BCP granules (blue dots).

Furthermore, we assessed the developed BCP granules as a potential platform for the delivery of antimicrobial agents based on the release of methylene blue (MB) selected as a modeling antibacterial drug from the BCP granules. The profile of MB release from the initial Mg-5BCP granules is presented in Figure 4e. According to these data, 95% of the total yield of MB was released during the first day. The release rate may have been decreased by encapsulating the methylene blue in a polymer matrix, in particular, methylcellulose (MC). The viscosity of the MC gel was expected to significantly affect the release rate; therefore, we studied the influence of temperature on the MC rheological behavior at 25 °C (preparation conditions) and 37 °C (physiological conditions) (Figure 4a,b). In this range, the viscosity of the 5% MC solution was gradually decreasing, which correlated with the previously reported data: the obtained T2 temperature was found at 50 °C [39]. The dependency of viscosity from concentration (Figure 4c,d) relates to the model of the gradual increase in the slope of the log(η)-log(c) graph due to the transition between the diluted, semi-diluted, and concentrated states of polymer chains [40]. The obtained transition point was calculated at 4.75 wt%, where the formation of the concentrated chain network occurred.

The release profile of MB encapsulated in the Mg-5BCP granules by the introduction of MC proved the influence of the viscous polymer matrix on the release prolongation. Only 75% of the total yield of MB was released on day 1 and nearly 5% was still retained in the composite for 3 days.

Based on the obtained data, MC-encapsulated Mg-5BCP can present a promising platform for further studies regarding antibacterial loading, aimed at prolonged release. While this study presents a proof-of-concept (single-replicate, limited discretion in the initial release time range), more research is necessary to precisely examine the early-phase burst release. Moreover, further studies may focus on the testing of the synergistic activity with additional antibacterial agents, as this may affect both release profiles and antibacterial performance [41].

**Figure 5:**
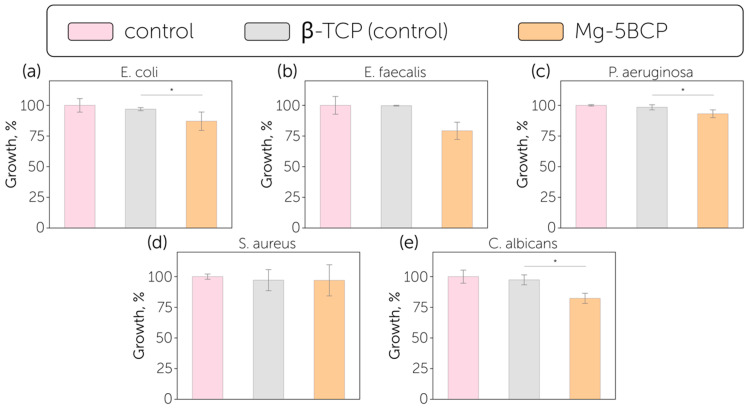
Growth percentage (bars) and standard deviation (SD) for (**a**) *E. coli*, (**b**) *E. faecalis*, (**c**) *P. aeruginosa*, (**d**) *S. aureus*, and (**e**) *C. albicans* cultured in the absence of substrates (pink), pure β-TCP (gray), and Mg-5BCP (orange) (n = 3). Data were obtained from three independent experiments and are expressed as mean percentage values ± SD, calculated relative to the growth observed in the presence of TCP and the blank control, both considered as the reference values and set at 100%. *p*-values, obtained using one-way ANOVA, are reported as follows: * = *p* < 0.05; compared to the positive control.

The antibacterial and antifungal activities of β-TCP and Mg-5BCP substrates were evaluated against five microorganisms (*E. coli*, *S. aureus*, *P. aeruginosa*, *E. faecalis*, and *C. albicans*) (Figure 5). In each experiment, a positive control was included, consisting of each microorganism cultured in the absence of substrates for 24 h at its optimal growth temperature. Growth percentages and corresponding standard deviations were determined from the mean optical density measurements at 600 nm (OD600) obtained from three independent replicates. The Mg-5BCP material exhibited promising antimicrobial activity against *C. albicans* (growth inhibition was 17.7 ± 4.1%), and *E. faecalis* (growth inhibition was 20.8 ± 7.0%) and modest results in inhibiting the growth of *E. coli* (growth inhibition was 12.9 ± 7.5%), while the inhibition of other tested bacterial models (*P. aeruginosa* and *S. aureus*) was negligible. It is worth noting that the inhibition efficiency for Mg-5BCP was significantly higher compared to the tricalcium phosphate control, which reached its maximum growth inhibition of 3.1 ± 1.3% on the *E. coli* model. Therefore, Mg-5BCP may further prolong the antibacterial background after an extensive release of an antibacterial drug during the first week.

Magnesium addition was reported to increase the solubility of calcium phosphate-based ceramics [42,43], which was accompanied by significantly higher antibacterial activity presented by Mg-5BCP compared to β-TCP, which correlated with the previous study [19]. Therefore, the increased solubility may have been the main reason for the rise in the antibacterial activity of both boron-contained and magnesium-contained calcium phosphate ceramics. Thus, a further increase in the solubility of the material or the formation of granules with larger surface areas may lead to even better antibacterial effect. Sinulingga et al. suggested two potential mechanisms of the metal cation-derived bacterial growth inhibition: the direct contact between the bacterial wall and a metal ion and the formation of reactive oxygen species, which lead to the disruption of the bacterial membrane [44]. Since the growth inhibition was significantly affected by the biodegradation rate, i.e., the concentration of metal ions on the media, the second mechanism apparently plays a more sufficient role.

Compared to less-soluble magnesium-substituted hydroxyapatite that showed a modest increase in the inhibition of *E. coli*, *S. aureus*, *P. aeruginosa*, *E. faecalis*, and *C. albicans* growth on non-substituted HAp, Mg-substituted BCP ensured a significant increase [45]. A similar effect was shown for HAp/MgO composites; however, the biodegradation rate of these phases was expected to be considerably different [46]. In addition, Mg-5BCP showed a comparable growth inhibition of *E. coli* with copper-substituted HAp and outperformed strontium-substituted HAp [44,47]. The dual Mg/Cu co-substitution for HAp showed higher inhibition degrees, so the possible synergetic effect was revealed, as magnesium and copper are expected to provide different inhibition mechanisms [48].

It is worth noting that, despite the promising antibacterial performance, Mg-substituted calcium phosphates were also reported to possess significant osteoinductive potential. In particular, Mg-doped biphasic calcium phosphate ceramics were shown to enhance osteogenesis (increase in osteogenesis-related gene expression) and improve angiogenesis [49]. Therefore, future studies may also be focused on the correlations between the BCP’s composition, including the magnesium content, and its osteoinductivity.

## 4. Conclusions

Magnesium content has been shown to affect the phase composition of powders obtained after the sintering of non-stoichiometric hydroxyapatite. In particular, low magnesium content (1%) led to the formation of hydroxyapatite as a primary phase, while high magnesium content resulted in the formation of tricalcium phosphate. Therefore, intermediate magnesium concentrations allowed for biphasic powders to be obtained; in particular, MgBCP with a 5% magnesium content was synthesized and its antibacterial performance has been assessed. Mg-5BCP showed promising results in inhibiting the growth of *C. albicans* (growth inhibition was 17.7 ± 4.1%), *E. faecalis* (growth inhibition was 20.8 ± 7.0%), and *E. coli* (growth inhibition was 12.9 ± 7.5%).

Moreover, Mg-5BCP-based porous granules were fabricated via the sodium alginate gelation stage with a subsequent burning out of the polymer to use as an antibacterial drug delivery platform. Loading of the model drug (methylene blue) to the obtained granules led to the burst release; therefore the encapsulation to the methylcellulose matrix was implemented, which resulted in a prolonged release.

## Data Availability

The original contributions presented in this study are included in the article. Further inquiries can be directed to the corresponding authors.

## Abbreviations

The following abbreviations are used in this manuscript:

Mg-BCP	Magnesium-substituted biphasic calcium phosphate
HAp	Hydroxyapatite
β-TCP	β-tricalcium phosphate
BCP	Biphasic calcium phosphate
MgHAp	Magnesium-substituted non-stoichiometric hydroxyapatite
SA	Sodium alginate
XRD	X-ray diffraction
MB	Methylene blue
MC	Methylcellulose
